# Seven-Year Surveillance of *emm* Types of Pediatric Group A Streptococcal Pharyngitis Isolates in Western Greece

**DOI:** 10.1371/journal.pone.0071558

**Published:** 2013-08-19

**Authors:** George A. Syrogiannopoulos, Ioanna N. Grivea, Adnan Al-Lahham, Maria Panagiotou, Alexandra G. Tsantouli, Aspasia N. Michoula Ralf René Reinert, Mark van der Linden

**Affiliations:** 1 University of Thessaly, School of Health Sciences, Faculty of Medicine, General University Hospital of Larissa, Biopolis, Larissa, Greece; 2 Institute for Medical Microbiology and National Reference Center for Streptococci, University Hospital, Aachen, Germany; 3 School of Applied Medical Sciences, German Jordanian University, Amman, Jordan; 4 University of Patras, School of Health Sciences, Faculty of Medicine, General University Hospital of Patras, Rion, Patras, Greece; Rockefeller University, United States of America

## Abstract

**Background:**

An experimental 26-valent M protein Group A streptococcal (GAS) vaccine has entered clinical studies. Pharyngeal GAS *emm* type surveillances in different areas and time-periods enhance the understanding of the epidemiology of GAS pharyngitis. Moreover, these surveillances, combined with the data on GAS invasive disease, can play a significant role in the formulation of multivalent type-specific vaccines.

**Methods:**

During a 7-year period (1999–2005), 2408 GAS isolates were recovered from consecutive children with pharyngitis in Western Greece. The overall macrolide resistance rate was 22.8%. Along the study period we noted a tendency towards significantly decreased rates of resistance, with the lowest rates occurring in 2002 (15.3%), 2003 (15%) and 2004 (16.7%). A random sample of isolates from each year, 338 (61.7%) of the 548 macrolide-resistant and 205 (11%) of the macrolide-susceptible, underwent molecular analysis, including *emm* typing.

**Results:**

The 543 typed isolates had 28 different *emm* types. A statistically significant association was found between macrolide resistance and *emm*4, *emm*22 and *emm*77, whereas *emm*1, *emm*3, *emm*6, *emm*12, *emm*87 and *emm*89 were associated with macrolide susceptibility. A significant yearly fluctuation was observed in *emm*4, *emm*28 and *emm*77. The most common macrolide-resistant GAS were *emm*77 isolates harboring *erm*(A), either alone or in combination with *mef*(A), *emm*4 carrying *mef*(A), *emm*28 possessing *erm*(B), *emm*75 carrying *mef*(A), *emm*12 harboring *mef*(A) and *emm*22 carrying *erm*(A). We estimated that 82.8% of the isolates belonged to *emm* types included in the novel 26-valent M protein vaccine. The vaccine coverage rate was determined mainly by the increased frequency of nonvaccine *emm*4 isolates.

**Conclusions:**

A limited number of *emm* types dominated among macrolide-susceptible and macrolide-resistant GAS isolates. We observed seasonal fluctuations, which were significant for *emm*4, *emm*28 and *emm*77. This type of data can serve as baseline information if the novel 26-valent M protein GAS vaccine is introduced into practice.

## Introduction

Group A streptococcal (GAS) infections are a major cause of morbidity and mortality worldwide. GAS pharyngitis is one of the most common bacterial infections in school age children. Furthermore, GAS also causes a variety of skin and soft tissue infections, severe invasive disease, toxin-associated syndromes and the nonsuppurative sequelae of acute rheumatic fever, acute glomerulonephritis and, probably, reactive arthritis [1].

The M protein, encoded by the *emm* gene, is considered a major virulence factor and the major immunologic epitope of GAS [2]. The M protein possesses a hypervariable region of the amino-terminal 40 to 50 amino acid residues [3]–[6]. A GAS typing system based on sequencing of this N-terminal hypervariable region of the M protein (*emm*) gene has been used for identification of different *emm* types. More than 150 types have been recognized worldwide to date [7], [8].

Small N-terminal M protein peptides evoke protective antibodies against epidemiologically important GAS serotypes with the greatest bactericidal activity and are least likely to cross-react with human tissues. This discovery enabled investigators to develop recombinant multivalent N-terminal type-specific vaccines [9], [10].

A multivalent vaccine encompassing small N-terminal M protein peptides from 26 different *emm* types has been developed and has entered clinical studies [11], [12]. The effectiveness of this M protein vaccine may be highly dependent on the *emm* type coverage of the clinical isolates. And, therefore, the *emm* type distribution may guide further vaccine development.

The situation in Europe has drawn attention because of the significant variation in *emm* type distribution that might exist in relation to the increased rates of macrolide-resistant GAS isolates that have been noted in some countries in late 1990 s and early 2000 s [13]–[16]. Pharyngeal GAS *emm* type systematic surveillance enhances the understanding of the epidemiology of pharyngitis GAS disease and the formulation of multivalent type-specific vaccines.

Greece is a European country with increased rate of macrolide-resistant GAS isolates [17], [18]. We established a primary care network in Western Greece, in order to prospectively study children with acute GAS pharyngitis in a systematic fashion. The aim of the present 7-year study was to investigate among pharyngeal GAS isolates (i) the phenotypes and genotypes of macrolide-resistant isolates, (ii) the *emm* type and subtype distribution and (iii) the proportion of isolates that could be covered by the 26-valent M protein-based GAS vaccine currently under clinical investigation.

## Materials and Methods

### Ethics Statement

The research protocol was approved by the Ethics Committee of the General University Hospital of Patras. A written informed consent was obtained from each child's parent or legal guardian. The data were analyzed anonymously.

### Subjects and specimens

Between January 1999 and December 2005, 2408 GAS isolates were recovered from consecutive children with pharyngitis living in various areas of Western Greece. The study was conducted in 9 sites in a total of 5 prefectures: Preveza (Preveza), Agrinio and Nafpaktos (Etoloakarnania), Patras and Egion (Achaia), Pyrgos and Amaliada (Ilia), and Kalamata and Gargaliani (Messinia). During the 7-year study period, the estimated average population of Western Greece per study year was 950.306 inhabitants, which included 174.061 children aged 0–16 years. The study population consisted of children 2–16 years old, with signs and symptoms of acute pharyngitis (fever, pharyngeal erythema and exudate, tender cervical lymph nodes, absence of conjunctivitis, rhinitis, hoarseness of voice or cough) confirmed by a positive throat culture for GAS. The study was performed in collaboration with 14 practicing pediatricians, who participate in our working group, the Hellenic Antibiotic-Resistant Respiratory Pathogens (HARP) Study Group. From November 2000 through December 2005, these pediatricians enrolled children with pharyngitis in clinical studies of different treatment regimens (Syrogiannopoulos GA, Grivea IN, Beratis NG, the HARP Study Group. 42^nd^ Intersci. Conf. Antimicrob. Agents Chemother., abstr. G-436, 2002; Syrogiannopoulos GA, Grivea IN, Kritikou D, the HARP Study Group. 43^rd^ Intersci. Conf. Antimicrob. Agents Chemother., abstr. G-1547, 2003; Syrogiannopoulos GA, Grivea IN, the HARP Study Group. 44^th^ Intersci. Conf. Antimicrob. Agents Chemother., abstr. G-2092, 2004; Syrogiannopoulos GA, Grivea IN, Chryssanthopoulou DC, Katopodis GD, the HARP Study Group. 46^th^ Intersci. Conf. Antimicrob. Agents Chemother., abstr. G-842, 2006) [19]. One GAS isolate from each child was included in the study. Throat cultures were performed by the same investigator (ING) initially at the Laboratory of the Division of Pediatric Infectious Disease of the University of Patras (1999–2004) and subsequently at the Laboratory of the Division of Pediatric Infectious Disease of the University of Thessaly (2004–2005).

Isolates were identified as GAS by typical colony morphology, β-hemolysis on sheep blood agar, Lancefield grouping, by using a commercially available agglutination technique (Slidex, Streptokit; BioMérieux, Marcy l′ Etoile, France), and by the pyrrolidonyl-arylamidase test.

GAS isolates were screened for susceptibility to erythromycin by both the disk diffusion method and the E test method (AB Biodisk, Sweden). The erythromycin-resistant GAS isolates were further studied for their antimicrobial susceptibility to erythromycin or clarithromycin by the broth microdilution method as described previously [17].

Over the 7-year study period, resistance to macrolides was found in 548 (22.8%) of the 2408 GAS isolates.

### Determination of macrolide resistance phenotypes

The macrolide resistance phenotypes, i.e. M, inducible MLS (iMLS) and constitutive MLS (cMLS), as well as their subtypes [20], were determined on the basis of the pattern of susceptibility to erythromycin and clindamycin and confirmed by the triple-disk (erythromycin plus clindamycin and josamycin) test. The triple-disk test was set up to facilitate the laboratory discrimination of the 3 subtypes (iMLS-A, iMLS-B, and iMLS-C) of the iMLS macrolide resistance phenotype, as described previously [20].

### Macrolide resistance determinants

GAS isolates showing resistance to erythromycin were tested by PCR for the presence of *erm*(A), *erm*(B), or *mef*(A) macrolide resistance determinants [21].

### *emm* Typed sample of macrolide-resistant GAS isolates

A representative sample of 338 (61.7%) of the 548 macrolide-resistant isolates was studied for their *emm* type and the presence of macrolide resistance determinants. The sample consisted of 181 (47.8%) of the 379 macrolide-resistant isolates collected during 1999–2002 and 157 (92.9%) of the 169 macrolide-resistant isolates from 2003–2005. For 1999–2002, the random selection was stratified by year taking into account the yearly number of isolates, the proportions of the different resistance phenotypes, the season and the study sites.

### *emm* Typed sample of macrolide-susceptible GAS isolates

Two hundred and five (11%) of the total 1860 macrolide-susceptible isolates were analyzed for their *emm* type. The random selection was stratified by year taking into account the yearly number of isolates, the season and the study site.

The isolates were studied for their *emm* type according to the method of Podbielski et al. [22]. Similarity searching was performed by using the N*-*terminal hypervariable region of the M gene based on the latest information from the Centers for Disease Control website (cdc.gov/ncidod/-biotech/strep/strains/emmtypes.html). GAS CS101 (*emm*49) was used as a reference strain. A limited number of isolates from 1999–2002 was *emm* typed and published previously [17].

### Statistical analysis

In each isolate a number was assigned and then, the numbers were entered into an array. Thereafter a CVF90 subtractive with a random generator number was applied to the array to select the isolates for *emm* type analysis. For the assessment of 2 groups, categorical parameters were compared using the Fisher's exact test. We independently assessed the difference between susceptible versus resistant isolates for a given *emm* type, thus we did not adjust for multiplicity [23]. Yearly fluctuation was tested using a log-linear model, with the year effect as exploratory variable and as response either the frequency of a certain *emm* type or the rate of macrolide-resistant isolates. Vaccine coverage was defined as the proportion of all isolates in the region that were covered by the 26-valent M protein-based GAS vaccine currently under clinical investigation [11]. The overall vaccine coverage was calculated as the weighted average of the two vaccine coverage rates: the one corresponding to the susceptible isolates and the one corresponding to the resistant isolates. We compared the vaccine coverage of the first versus the second period using z-test. Two-sided tests were used. The statistical analysis was performed using SPSS version 13.0 (SPSS Inc., Chicago, Ill). An effect was considered significant when *P*<0.05.

## Results

### Pharyngeal GAS isolates

**Figure 1:**
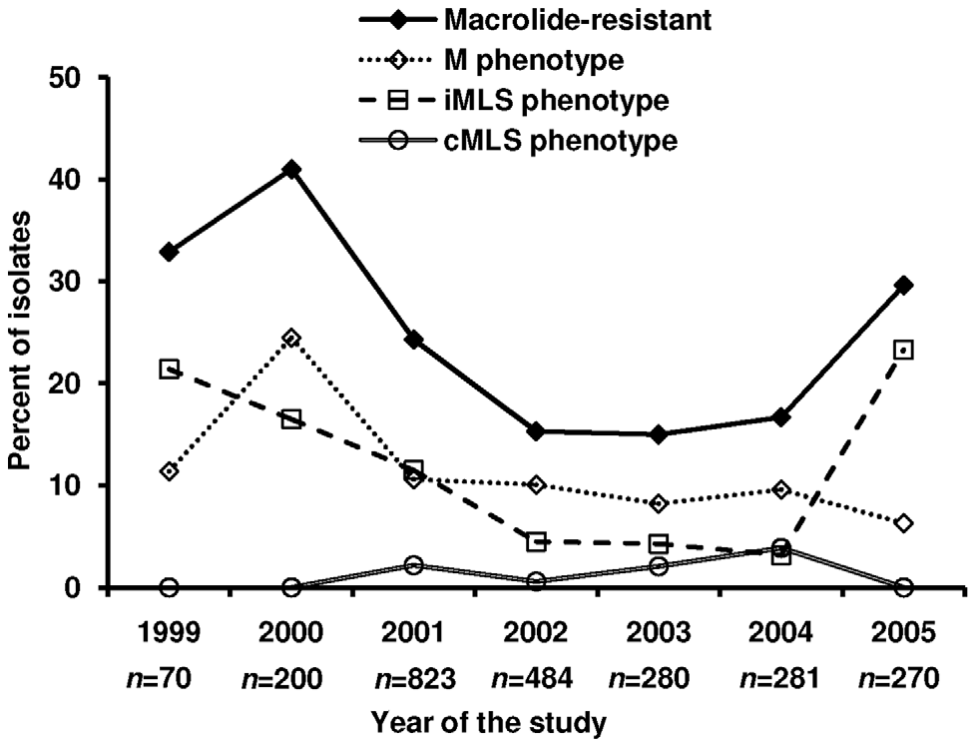
Macrolide resistance rate and resistance phenotypes among 2408 group A streptococcal isolates in Western Greece.

During the 7-year study period, 2408 GAS isolates were recovered from children with pharyngitis. GAS infections were mainly seen among children 5 to 10 years of age (79.4% of cases); the median age was 7 years. The number of GAS isolates gathered yearly, the rate of macrolide resistance and the resistance phenotypes appear in Figure 1. Resistance to macrolides was found in 548 (22.8%) of the 2408 isolates. More than half (52.6%) of these macrolide-resistant isolates exhibited either the inducible or the constitutive MLS phenotype. Specifically, 47.4% had the M phenotype, 0.7% the iMLS-A, 18.3% had the iMLS-B, 26.5% the iMLS-C, and finally 7.1% had the cMLS phenotype.

Along the study period we noted a tendency towards significantly decreased rates of resistance (*P*<0.001). The lowest rates occurred in 2002 (15.3%), 2003 (15%) and 2004 (16.7%). In 2005, an increase of isolates exhibiting the iMLS phenotype was noted; 92% had the iMLS-B subtype, 4.8% the iMLS-C and 3.2% the iMLS-A.

A random sample of 543 isolates, 205 macrolide-susceptible and 338 macrolide-resistant, was further analyzed with resistance genotyping and *emm* typing. Of the 338 macrolide-resistant isolates, 43.5% had the M phenotype, 1.2% the iMLS-A, 23.1% showed the iMLS-B, 21.6% the iMLS-C, and finally 10.6% had the cMLS phenotype. There was no significant difference in the frequency of any given phenotype in the typed sample of macrolide-resistant isolates compared to that found in the total collection of macrolide-resistant isolates. The 338 typed macrolide-resistant isolates harbored the *mef*(A) (43.7%), *erm*(A) (36.1%), *erm*(A) plus *mef*(A) (8.9%), *erm*(B) (8.6%) or *erm*(B) plus *mef*(A) (2.7%) gene. A combination of macrolide resistance determinants was revealed in 11.5% of macrolide-resistant isolates.

### *emm* Types of GAS isolates

The 543 typed isolates belonged to 28 different *emm* types; the macrolide-susceptible belonged to 25 types and the macrolide-resistant to 18 (Table 1). A statistically significant association was found between macrolide resistance and *emm*4, *emm*22 and *emm*77, whereas *emm*1, *emm*3, *emm*6, *emm*12, *emm*87 and *emm*89 were associated with macrolide susceptibility. However, there were 2 distinct groups of isolates regarding their association with macrolide resistance or susceptibility. In the one group of *emm* types, *emm*4, *emm*12, *emm*22 and *emm*77, were observed populations of both macrolide-resistant and -susceptible isolates. In contrast, in the other group of *emm* types, *emm*3, *emm*6 and *emm*87, macrolide-resistant isolates were rare.

**Table 1:** *emm* type distribution of the macrolide-susceptible and macrolide-resistant group A streptococcal isolates.

*emm* type	Macrolide- susceptible	Macrolide- resistant	*P*
	(n = 205)	(n = 338)	
12	44 (21.5)^a^	25 (7.4)	**<0.001**
1	28 (13.7)	9 (2.7)	**<0.001**
77	19 (9.3)	100 (29.6)	**<0.001**
89	15 (7.3)	11 (3.3)	**0.038**
3	13 (6.3)	1 (0.3)	**<0.001**
6	13 (6.3)	2 (0.6)	**<0.001**
28	13 (6.3)	26 (7.7)	0.610
2	11 (5.4)	11 (3.3)	0.264
4	10 (4.9)	98 (29)	**<0.001**
11	10 (4.9)	9 (2.7)	0.228
75	7 (3.4)	19 (5.6)	0.302
87	5 (2.4)	1 (0.3)	**0.031**
22	3 (1.5)	21 (6.2)	**0.009**
Other	14^b^ (6.8)	5^c^ (1.5)	**0.001**

a Number in parentheses, percent.

b *emm* type (no. of isolates): 23 (1), 25 (1), 29 (2), 44 (1), 50 (1), 65 (1), 68 (1), 102 (1), 110 (2), 118 (1), PT3875 (1), ST3211 (1).

c *emm* type (no. of isolates): 15 (1), 25 (1), 49 (1), 102 (1), 106 (1).

Among the macrolide-susceptible isolates, 4 *emm* types, *emm*12, *emm*1, *emm*77 and *emm*89, accounted for 51.7% of isolates, while 7 types, *emm*12, *emm*1, *emm*77, *emm*89, *emm*3, *emm*6 and *emm*28, accounted for 70.7% and the 10 most prevalent for 85.9% of isolates (Table 1).

Macrolide-resistant isolates belonged to a limited number of *emm* types. Specifically, *emm*4, *emm*75, *emm*12 and *emm*1 accounted for 72.3% of the erythromycin-resistant GAS isolates harboring *mef*(A) as the sole macrolide resistance determinant (Table 2). Furthermore, *emm*77, *emm*4 and *emm*22 accounted for 77% of those carrying *erm*(A), either alone or in combination with *mef*(A), whereas *emm*28 and *emm*12 accounted for 57.9% of the possessing *erm*(B) isolates, either alone or in combination with *mef*(A*).*

**Table 2:** *emm* type distribution of the 338 macrolide-resistant group A streptococcal isolates with different macrolide resistance genotypes.

			Macrolide resistance genotypes				
*emm* type		No. of isolates	*mef*(A)	*erm*(A)	*erm*(A) plus *mef*(A)	*erm*(B)	*erm*(B) plus *mef*(A)
77	100		13 (13)^a^	62 (62)	24 (24)	0	1 (1)
4	98		74 (75.5)	13 (13.3)	5 (5.1)	2 (2)	4 (4.1)
28	26		7 (26.9)	3 (11.5)	0	16 (61.5)	0
12	25		13 (52)	6 (24)	0	5 (20)	1 (4)
22	21		6 (28.6)	13 (61.9)	0	1 (4.8)	1 (4.8)
75	19		16 (84.2)	2 (10.5)	0	1 (5.3)	0
2	11		0	11 (100)	0	0	0
89	11		5 (45.5)	3 (27.3)	0	2 (18.2)	1 (9.1)
1	9		4 (44.4)	4 (44.4)	0	0	1 (11.1)
11	9		5 (55.6)	2 (22.2)	0	2 (22.2)	0
Other	9		5^b^ (55.6)	3^c^ (33.3)	1^d^ (11.1)	0	0

a Number in parentheses, percent.

b *emm* type (no. of isolates): 3 (1), 6 (1), 49 (1), 102 (1), 106 (1).

c *emm* type (no. of isolates): 6 (1), 15 (1), 87 (1).

d *emm* type (no. of isolates): 25 (1).

**Figure 2:**
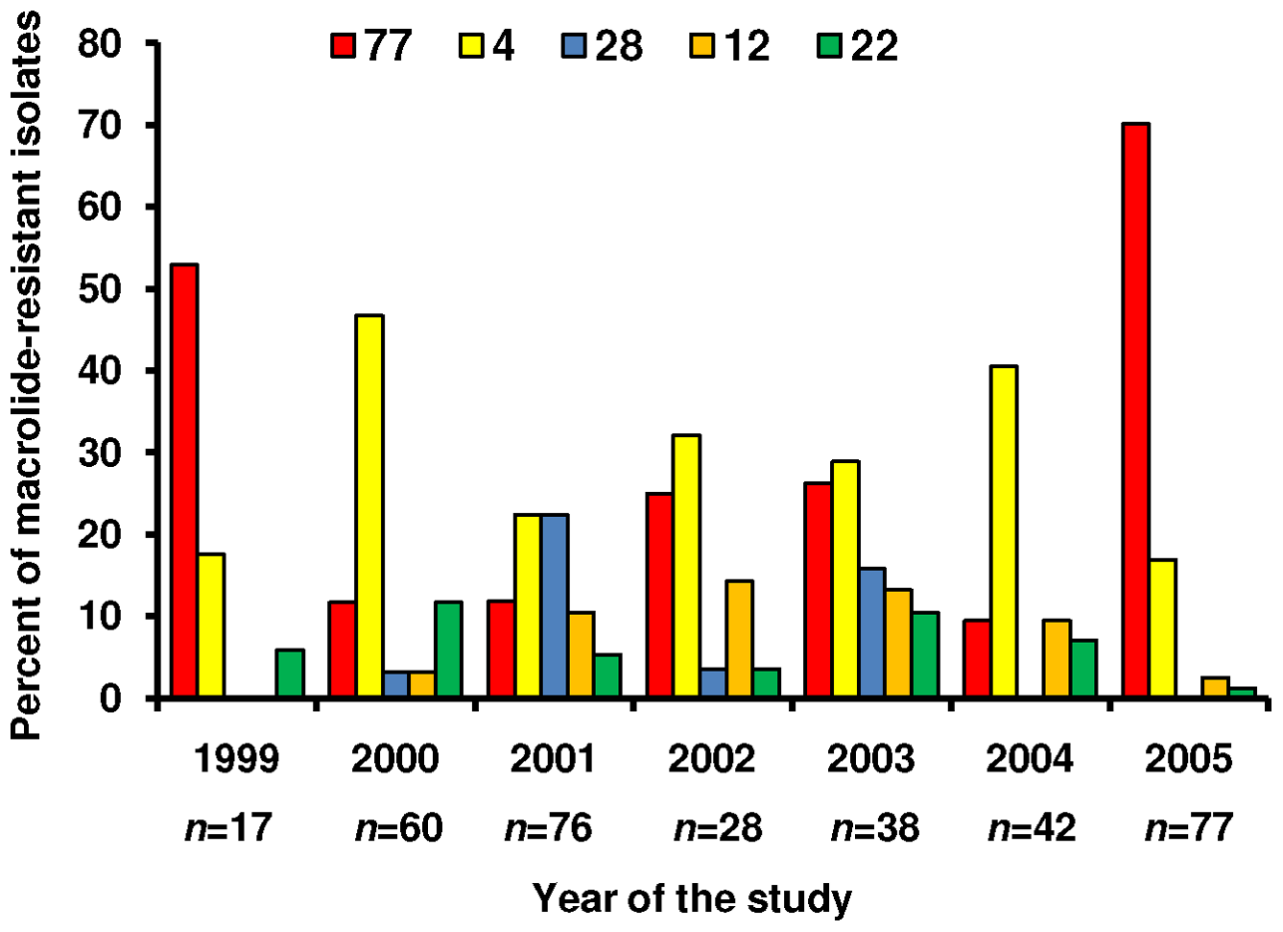
Macrolide-resistant group A streptococcal isolates. Annual frequencies of the 5 most common *emm* types. A significant yearly variation was observed in *emm*28 (*P*<0.001), *emm*77 (*P*<0.001) and *emm*4 (*P*<0.005).

There was seasonal fluctuation in the predominant *emm* types. Among macrolide-susceptible isolates, the variation in those belonging to *emm*28 and *emm*77 reached a statistically significant level (*P*<0.01). On the other hand, among macrolide-resistant isolates, a significant seasonal fluctuation was observed in those belonging to *emm*4, *emm*28 and *emm*77 (Figure 2). In 2005, we noted an increased number of GAS isolates belonging to *emm*77, expressing the iMLS phenotype. They all exhibited the iMLS-B subtype and harboured *erm*(A), either alone or in combination with *mef*(A).

### Prevalence of the classic rheumatogenic types (*emm*3, *emm*5, *emm*6, *emm*14, *emm*18, *emm*19 and *emm*29)

Of the 543 typed pharyngeal isolates, only two belonged to *emm*29 and none to *emm*5, *emm*14, *emm*18, and *emm*19. In addition, *emm*3 and *emm*6 made up to 6.3% of macrolide-susceptible isolates each, while they represented 0.3% and 0.6% of macrolide-resistant isolates, respectively.

### Subtypes

Among the 10 most prevalent *emm* types, we observed considerable variability in the frequencies of the respective subtypes. We identified 12 *emm* subtypes: *emm*1.14, *emm*1.37, *emm*3.1, *emm*3.35, *emm*6.4, *emm*6.54, *emm*12.11, *emm*12.21, *emm*12.23, *emm*12.37, *emm*28.5, *emm*29.4. Within *emm*1, *emm*3, *emm*6, *emm*12, *emm*28 and *emm*29 isolates, there were 1–4 subtypes, accounting for 2.6%–50% of all isolates of a given *emm* type.

### *emm* Types of the representative isolates in relation to an experimental 26-valent GAS vaccine

**Figure 3:**
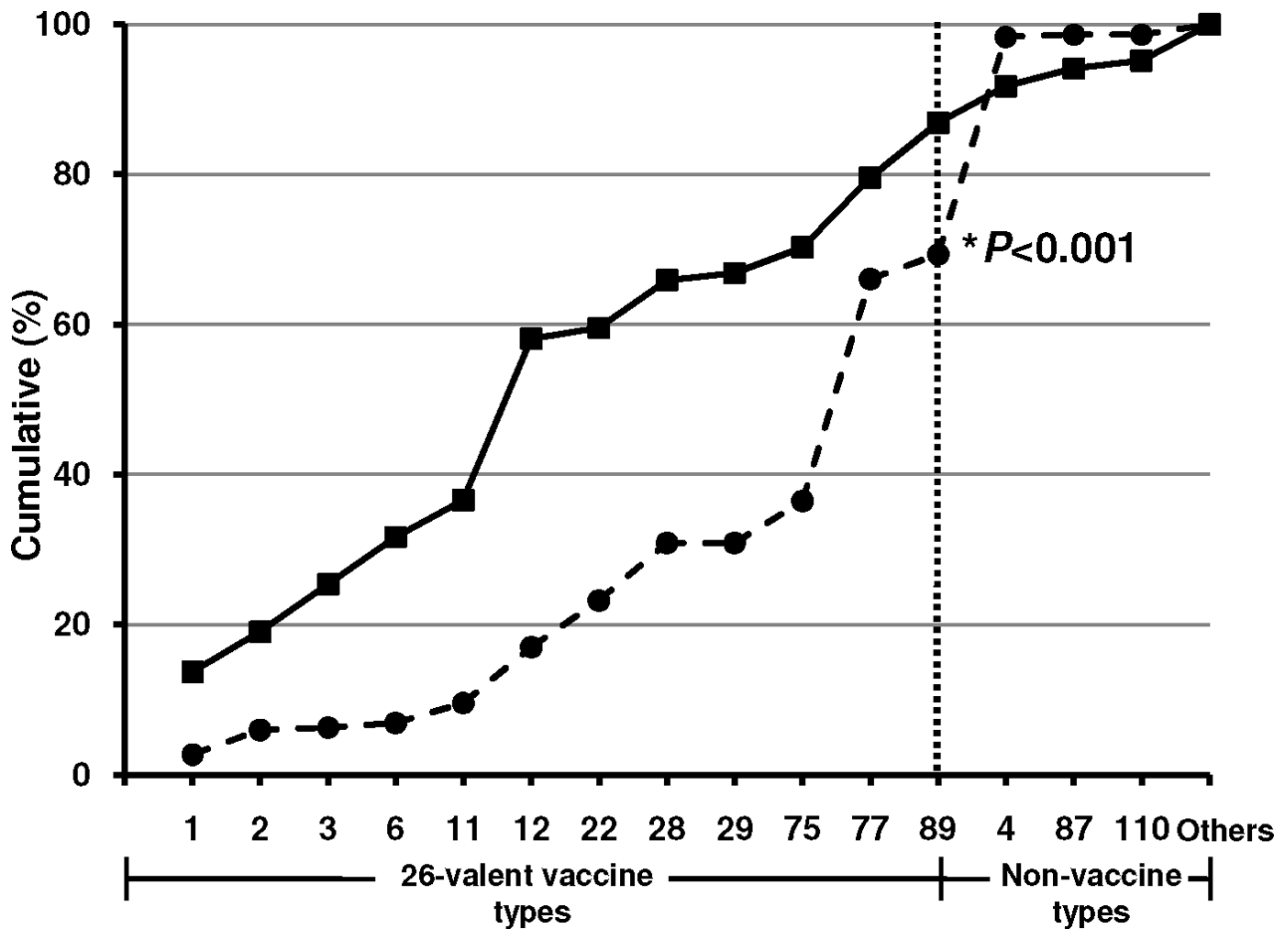
Cumulative distribution of the *emm* types of the macrolide-susceptible (solid line) and the macrolide-resistant (dashed line) group A streptococcal (GAS) isolates, according to the types included in an experimental 26-valent GAS vaccine.

Twelve of the 28 *emm* types found in Greek pharyngeal isolates are included in an experimental 26-valent GAS vaccine. The cumulative distributions of *emm* types of macrolide-susceptible and macrolide-resistant isolates are presented in Figure 3. We found that 178 (86.8%) of the 205 macrolide-susceptible isolates and 234 (69.2%) of the 338 macrolide-resistant isolates belonged to *emm* types that are included in the vaccine (*P*<0.001). The lower coverage of macrolide-resistant isolates was attributed to 7 nonvaccine *emm* types, *emm*4 (predominant), *emm*15, *emm*25, *emm*49, *emm*87, *emm*102 and *emm*106.

Among macrolide-susceptible isolates, the vaccine coverage was 84.9% for 1999–2002 vs. 90.8% for 2003–2005 (z-test = 1.204, *P = *0.277). On the other hand, among macrolide-resistant isolates, the vaccine coverage was 65.7% for 1999–2002 vs. 73.2% for 2003–2005 (z-test  = 1.5, *P = *0.157).

### Estimated overall coverage rate by an experimental 26-valent M protein GAS vaccine

We applied the coverage rate observed among the representative macrolide-susceptible isolates and the one found among the representative macrolide-resistant isolates in order to estimate the overall coverage rate in the total collection of macrolide-susceptible and macrolide-resistant isolates gathered over the 7-year study.

Total number of macrolide-susceptible isolates x coverage rate among macrolide-susceptible isolates (%) + total number of macrolide-resistant isolates x coverage rate among macrolide-resistant isolates (%)/total number of isolates.

## Discussion

We assessed temporal trends in GAS *emm* type distribution among pediatric pharyngeal isolates collected systematically from 9 scattered geographic sites in Western Greece over a 7-year period. Special attention has been paid to macrolide-resistant isolates. This is one of the largest published collections of *emm* typed macrolide-resistant isolates along with a random sample of macrolide-susceptible isolates recovered from children with pharyngitis. Several features are of importance in this prospective study: number of patients enrolled (over 2400), duration of the observation (7 years), homogeneity of patients enrolled in terms of age (2–16 years) and of clinical presentation, same investigators and same methodology throughout the study period.

In Greece from 1990 to 2005, macrolide and lincosamide consumption has been substantially increased, with the highest consumption rate noted in 2004 (2005 IMS Health database) [24]. Among GAS isolates circulating in Western Greece, there was an initial phase of a prominent increase in the rate of macrolide resistance, 1999–2000, followed by a second phase, initiated in 2001, characterized by a significant trend of decline in the resistance rate. Increased rates of macrolide-resistant GAS, sometimes as high as 35%, followed by a decrease have been also reported in recent studies from other European countries [25], [26]. It has been suggested that this decline may be attributed to decreased macrolide consumption [27] as well as GAS clonal properties [28]. Even though there has been a decrease in the rates of macrolide resistance among GAS isolates, it should be underlined that Greece remains one of the countries with the highest macrolide resistance rates in Europe. Specifically, the rate of macrolide-resistant GAS isolates ranged from 18.8% in Athens, Greece, during 2006 [29] to 24% in Central Greece, between January 2007 and June 2009 [18].

In this study, a statistically significant association was found between macrolide susceptibility and *emm*1, *emm*3, *emm*6, *emm*12, *emm*87 and *emm*89. In previous reports, a similar correlation was found between macrolide susceptibility and *emm*1 [29], [30], *emm*3 [28], [30], *emm*12 [30], *emm*6 [28] and *emm*89 [28]. On the other hand, we also established significant association between macrolide resistance and *emm*4, *emm*22 and *emm*77. In previous studies, a similar correlation has been found for *emm*4 [28], [30]–[32], *emm*22 [28], [29], [31] and *emm*77 [29].

Although we observed considerable diversity in *emm* types among macrolide-susceptible GAS isolates, relatively few types dominated. The 7 most prominent *emm* types were *emm*12, *emm*1, *emm*77, *emm*89, *emm*3, *emm*6 and *emm*28 (in descending order) and accounted for 70.7% of susceptible isolates. Similarly, in a 7-year surveillance of pediatric pharyngitis isolates from North America, these 7 *emm* types were among the 10 most common *emm* types in the United States, accounting for 67.1% of isolates, and among the 9 most common *emm* types in Canada, accounting for 68.6% of isolates [33]. In this large surveillance, the prevalence was estimated on the basis of the total number of isolates and not according to macrolide susceptibility. It should be underlined that the macrolide resistance rates in these countries are significantly lower compared to those observed in our study [34]. Furthermore, a similar dominance by relatively few types has been reported in studies of pharyngeal isolates from Ontario, Canada [35] and Western European countries, such as Sweden [36].

The present study showed that the majority of macrolide-resistant isolates belonged to a limited number of *emm* types. *emm*4, *emm*75, *emm*12, and *emm*1 accounted for 72.3% of the erythromycin-resistant GAS isolates harboring *mef*(A) as the sole macrolide resistance determinant. The same types accounted for 68.2%, 77.1%, and 100% of *mef*(A)-positive macrolide-resistant GAS isolates in Italy, North America, and France, respectively [13], [34], [37]. *erm*(A), either alone or in combination with *mef*(A), was carried mainly by *emm*77 isolates. The same type accounted for 100% of GAS isolates possessing *erm*(A) in an study in Italy [37]. Moreover, Zampaloni et al. [31] studied the macrolide resistance phenotypes, including subtypes, and reported that 12 (92.3%) of the 13 isolates exhibiting the iMLS-B or iMLS-C belonged to *emm*77. Finally, in the present study, *emm*28 was found to be predominant among the *erm*(B)-positive macrolide-resistant GAS isolates. The same type accounted for 50% and 70% of the *erm*(B)-positive macrolide-resistant GAS isolates in the United States and France, respectively [13], [34].

Our 7-year survey revealed a marked prevalence of macrolide-resistant GAS isolates belonging to *emm*4. The frequency of *emm*4 was 5.9-fold higher among macrolide-resistant GAS compared to macrolide-susceptible isolates. Increased prevalence of *emm*4 has been identified in a recent survey from Ontario, Canada [35] as well as in other pharyngitis surveys [28], [33], [36]. A high frequency of *emm*4 among macrolide-resistant GAS isolates, mostly with M phenotype, has been described in Spain (ranked 1^st^) [30], in Italy (2^nd^ marginally) [31], in Portugal (2^nd^) [28] and in the United States (3^rd^) [34].

*emm*77 was ranked third among our macrolide-susceptible GAS isolates and first among macrolide-resistant. However, it was found to have a rather low frequency in a thorough review of the available global data on GAS epidemiology [38].

This survey clearly demonstrates fluctuation in the frequency of pharyngeal *emm* types from season to season in Western Greece. A significant fluctuation was observed in *emm*4, *emm*28 and *emm*77. A recent study from Canada reported yearly fluctuations for *emm*1, *emm*3, *emm*28 and *emm*77 [35]. It has been postulated that the increased level of immunity to certain predominant types contributes to the decreased prevalence of some of these types in subsequent years [33]. Furthermore, it has been reported that several of the most common *emm* types account for a progressively lower proportion of pharyngeal isolates among older children and adolescents compared to younger children, suggesting a role for type-specific immunity [39]. In addition, it has been reported that serum antibody levels to several common M proteins were higher in older than in younger children [40].

Our data show that the classic rheumatogenic types had decreased prevalence, and in some instances completely disappeared. This decrease parallels the marked decline in incidence of acute rheumatic fever in our country [41].

In Western Greece during the study period, the 26-valent GAS vaccine coverage rate was estimated to be 82.8%; *emm*4 was the most common nonvaccine type. This coverage rate is close to that reported in recent studies on pharyngitis from North America. In a recent study of pediatric pharyngitis from the USA and Canada, the 26-valent vaccine coverage was approximately 85% [33]. In a study from Ontario, Canada, the 26-valent GAS vaccine coverage was 78.5% for pharyngitis cases, mean age 16.1 years, range 8 months to 105 years [35]. The vaccine coverage is higher in GAS pharyngitis rather than in invasive disease, which represents a major target for GAS immunization [42], [43]. Furthermore, it is higher in Established Market Economy countries than in lower income countries [38]. Lower coverage rates have been noted in other parts of the world, such as New Zealand [44].

It is thought that the circulation of macrolide-resistant isolates belonging to a common *emm* type, such as *emm*4, may be enhanced if a vaccine not including this type is used. *emm*4 has been included in a new 30-valent M protein-based GAS vaccine, which has entered the pre-clinical phase of evaluation [45].

A limitation of the present study may be the fact that we did not *emm* type a larger number or even the total number of macrolide-susceptible isolates. However, when we evaluated the typed macrolide-susceptible and macrolide-resistant isolates according to two time-periods, i.e. 1999–2002 compared to 2003–2005, we did not find a significant difference in their respective vaccine coverage rates.

In conclusion, a limited number of *emm* types dominated among macrolide-susceptible and macrolide-resistant GAS isolates recovered from consecutive children with pharyngitis in Western Greece during 1999–2005. A statistically significant association was found between macrolide resistance and *emm*4, *emm*22 and *emm*77, whereas *emm*1, *emm*3, *emm*6, *emm*12, *emm*87 and *emm*89 were associated with macrolide susceptibility. We observed seasonal fluctuations, which were significant for *emm*4, *emm*28 and *emm*77. We estimated that 82.8% of the pharyngeal isolates belonged to *emm* types that are included in an experimental 26-valent M protein vaccine. *emm*4 was the most common nonvaccine type. The molecular epidemiology data on macrolide-resistant and macrolide-susceptible GAS isolates in different time-periods and various areas, especially those with high prevalence of macrolide-resistant GAS, can serve as baseline information if the novel 26-valent M protein GAS vaccine is introduced into practice.
